# A 9-day-old neonate with giant scalp abscess: A case report

**DOI:** 10.1097/MD.0000000000017830

**Published:** 2019-11-27

**Authors:** Hongyuan Liu, Zongping Li, Liling Yang, Xu Yang, Yan Zhang, Jia Chen

**Affiliations:** a Department of Neurosurgery; b Department of Nephrology; c Department of Paediatrics, Mianyang Central Hospital, Mianyang, Sichuan, PR China.

**Keywords:** neonate, scalp abscess, scalp defect, scalp hematoma

## Abstract

**Rationale:**

Neonatal scalp mass is common in clinical practice. After birth canal compression and traction force, a cephalohematoma is usually found. However, cephalohematoma with abscess is extremely rare and dangerous. So far, there have been no reported cases of multidrug-resistant *Escherichia coli* infections in giant neonatal scalp hematoma.

**Patient concerns:**

We present a 9-day-old with a scalp abscess and a large scalp defect that remained after surgical drainage.

**Diagnosis:**

Physical examination showed a giant mass suggestive in the parietal region. B-mode ultrasound indicated the scalp mass was liquid. The early diagnosis was massive scalp hematoma. During conservative treatment, purulent fluid flowed from the mass region through a rupture in the scalp. MR examination showed the scalp had burst and no abnormalities were found in the medial side of the skull and skull.

**Interventions:**

The surgeon opened up the mass and removed necrotic tissue. The scalp was severely damaged; the aseptic auxiliary materials that we made in-house were used to gradually reduce the defect.

**Outcomes:**

The scalp was healed by anti-infection treatment and frequent changing of the dressings. The patient was successfully treated without two-stage surgery. There were no complications.

**Lessons:**

A scalp hematoma is a potential site of infection. Anti-infection treatment and surgery are necessary to correct infected scalp hematoma. This work offers a new way of treating other large scalp defects.

## 1 Introduction

Neonatal sepsis associated with microorganisms from the mother can be lethal.^[1]^*Escherichia coli*, which can spread to the blood, brain, soft tissue, and bones, is the most common causative pathogen of neonatal sepsis.^[2]^ The risks of neonatal infection include low gestational age, inhaled amniotic fluid, and intrapartum fever. Neonatal scalp mass is common in clinical practice. A cephalohematoma is often found after birth canal compression and tractive fetus.^[3]^ Abscess following infection of scalp hematoma is extremely rare. The factors affecting infection include direct hematogenous seeding, scalp contusion, and fetal monitoring. Surgery and anti-infection treatment are necessary for abscess. Here, we present an infected scalp hematoma with abscess formation and conservative treatment of large scalp defects in a full-term infant.

## 2 Case presentation

Written informed consent was obtained from the parents of the patient for publication of this case report and any accompanying images. A copy of the written consent form is available for review by the editor of this journal.

**Figure 1:**
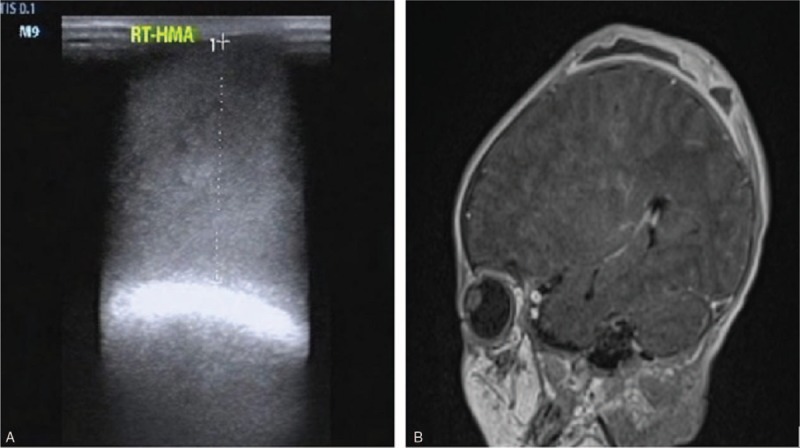
(A) B-mode ultrasound indicated the scalp mass was liquid and about 10.8 × 11.2 × 2.6 cm in size. (B) Enhanced MR examination, scalp had burst and no abnormalities were found in the medial side of the skull and skull.

**Figure 2:**
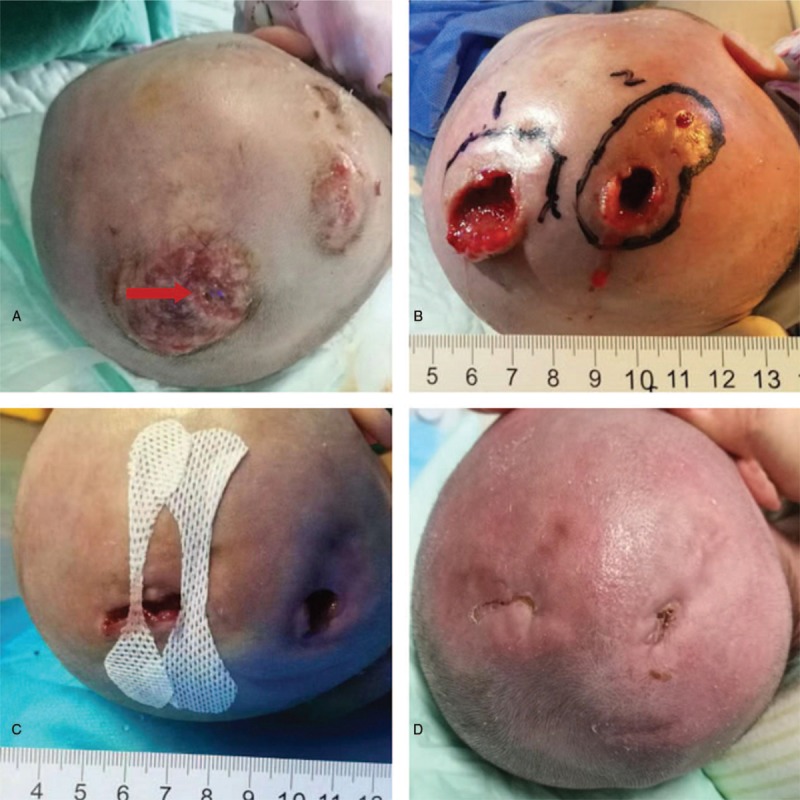
(A) At 9 days after birth, pus burst out from the discolored skin. (B) Two major scalp defects appeared, 3 × 2.5 × 0.5 cm and 1.5 × 1.0 × 0.7 cm in size. (C) Self-made aseptic auxiliary materials for changing the dressing were used on the large scalp defect. (D) The patient was successfully treated without the need for two-stage surgery. There were no complications.

A full-term infant girl was born by spontaneous vaginal delivery to a 27-year-old mother in a rural hospital. The infant's birth weight was 3.02 kg. After birth, a giant mass was observed over the parietal area. For treatment of the scalp mass, she was transferred to Mianyang Central Hospital. There was no intrauterine distress or premature rupture of membranes in prenatal examination. Breathing, amniotic fluid, umbilical cord, and placenta showed no abnormalities intrapartum. APGAR scores were unknown. A mass about 10 × 10 cm in size was found over the parietal area, and there was no scalp wound. The mass was soft, elastic, and high tension, not extending beyond the cranial suture. Blood analysis and coagulation function were within normal limits. B-mode ultrasound indicated the scalp mass was liquid, the size was about 10.8 × 11.2 × 2.6 cm (Fig. 1A). Blood Rt and C-reactive protein (CRP) was normal. In the process of observation, fever (T, 38.2°C) appeared on Day 3 of life. Blood examination indicated that CRP was 57.5 mg/L, white blood cell (WBC) count was 10.76 × 10^9^/L, neutrophilic granulocyte percentage (NEUT%) was 72.1%, procalcitonin (PCT) was 0.3 μg/L, but the blood culture was not back. Chest radiography showed changes to the lung texture and enlargement of the lungs. Fever was considered for neonatal pneumonia or hematoma absorption. Cefuroxime sodium (25 mg/kg every 8 hours) was administered by intravenous infusion. Polypnea developed and fever (T40°C) persisted on Day 5 of life, physical and drug for symptomatic treatment. WBC count was 18.74 × 10^9^/L, CRP increased to 163 mg/L, PCT was 1.5 μg/L, and the results of lumbar puncture were normal. *E coli* that extended-spectrum β-lactamases was positive resistance to cefuroxime sodium and levofloxacin and sensitive to piperacillin sulbactam and meropenem, was detected in blood culture and sputum. Chest radiography showed changes in lung texture and lung enlargement. Biochemical examination was performed on the puncture fluid of the mass. The skin of the mass became discolored locally during anti-infective therapy. Piperacillin sulbactam (280 mg every 6 hours) was administered by intravenous infusion. On the second day after administration of antibiotics, the body temperature dropped. At 9 days after birth, pus burst out from the discolored skin (Fig. 2A), the body temperature increased to 40.2°C. To prevent exposing the patient to excessive radiation, a magnetic resonance imaging (MRI) scan was performed. Results showed the scalp had burst. They showed no abnormalities in the medial side of the skull and skull (Fig. 1B). The results of lumbar puncture were normal. *E coli* was detected in the pus and puncture fluid cultured. The scalp abscess was cut open, local necrotic tissue was removed, and pus was cultured before and after the surgery. Culture indicated *Escherichia coli* showed the same antibiotic sensitivity as the isolate from the blood culture. After the operation, there were 2 major scalp defects, 3 × 2.5 × 0.5 cm and 1.5 × 1.0 × 0.7 cm (Fig. 2B) in size. At the time of drainage and anti-infection treatment, the scalp defect was pulled using aseptic auxiliary materials that we made in-house to gradually reduce the defect (Fig. 2C). After 16 days of treatment, the patient was successfully treated without two-stage surgery. There were no complications (Fig. 2D).

## 3 Discussion

Scalp hematoma occurs most commonly after blunt instrument injury, premature delivery, primigravida delivery, or error by a midwife. The body often reabsorbs a scalp hematoma without intervention, but some large hematomas require puncture extraction and compression bandaging. A few hematomas need to be cut open to remove the hematoma and stanch the flow of any fluids. Neonatal scalp hematoma can be complicated by anemia, hypovolemic shock, infection, and other problems. In the absence of such risk factors as aspiration, scalp contusion, and fetal monitoring, bacterial infections in giant neonatal scalp hematoma are extremely rare. Currently, only one case of infected scalp hematoma associated with sepsis in neonates has been reported, and the *E coli* was not multi-drug resistant.^[4]^ In this paper, fever appeared on the third day after birth, and blood culture detected multidrug-resistant *E coli* was found in skin ulceration secretion culture and surgical incision of pus culture on Day 9 of life. These suggest that the blood-borne pathogen infection and sepsis are closely associated with large abscesses of the scalp. The scalp abscess is usually confined to scalp tissue but it may be accompanied by osteomyelitis or intracranial infection.^[5,6]^ MR and lumbar puncture examinations were normal. Hematomas can sometimes combine abscesses, so a culture should be performed of any secretions or puncture fluid.^[7]^ Due to past overuse of antibiotics in China, there are more and more superbugs infecting patients. It is very important to select antibiotics according to the culture results.^[8]^ Although the structures of subcutaneous abscesses, subgaleal abscesses, and subperiosteal abscesses are different, surgical debridement and sensitive antibiotics are key to treatment.

Scalp defects are a common complication after debridement of scalp abscess. There has been one case reported of scalp abscess associated with sepsis. This report did not list the size of the scalp defect or any subsequent treatment.^[4]^ Due to the special dome morphology of the scalp, methods of scalp reconstruction mainly include direct suturing, scalp expansion, and free flap.^[2,9,10]^ Since direct suturing requires a wide separation of loose connective tissue, most scalp defects cannot be sutured directly like skin defects in other parts of the body, except for a small number of small avulsion wounds. Tissue expanders are one way to treat large-area skin defects. Scalp expansion has the disadvantages of local skin necrosis, dilator exposure, local infection, and insufficient skin expansion, and treatment may require multiple surgeries.^[11]^ Free flap can be highly traumatic, time-consuming, and costly.^[9]^ In this paper, we describe a new method that is simpler, involves less pain, and reduces expenses. When changing fresh dressing for draining abscess, sterile auxiliary material is used to pull on the skin around the defect and reduce its area and ultimately repair the skin. This technique significantly shortens the treatment cycle, and reduces the need for unnecessary and expensive medical treatment.

## Author contributions

**Conceptualization:** Zongping Li.

**Investigation:** Liling Yang, Xu Yang.

**Methodology:** Hongyuan Liu.

**Project administration:** Zongping Li.

**Resources:** Yan Zhang, Jia Chen.

**Supervision:** Jia Chen.

**Validation:** Yan Zhang.

**Writing – original draft:** Hongyuan Liu, Xu Yang, Jia Chen.

**Writing – review & editing:** Hongyuan Liu.

Hongyuan Liu orcid: 0000-0001-9580-9715.
